# Supplementary material for: Effects of Substitution of Higher-Alcohol Products with Lower-Alcohol Products on Population-Level Alcohol Purchases: ARIMA Analyses of Spanish Household Data
Source: Nutrients. 2022 Oct 10;14(19):4209. doi: 10.3390/nu14194209 (PMC9572185; doi:10.3390/nu14194209)
Supplement: Supplementary file 1 [file nutrients-14-04209-s001.zip › nutrients-1933856-supplementary.pdf]

Supplement Table S1 Divisions of households by socio-demographic characteristics. (N=18,954 households).

Group	GDP per capita (€)	Categories	Number of households within each group	Distribution within each group (%)
Age of the main shopper (years)		18-34	3668	19.4
		35-49	7690	40.6
		50-64	5634	29.7
		65+	1962	10.4
Social grade[a]		Medium-High and High	3397	17.9
		Medium-Medium	6375	33.6
		Medium-Low	5081	26.8
		Low	4101	21.6
Autonomous Community	19,107	Andalusia	3319	17.5
	28,151	Aragon	547	2.9
	22,789	Asturias	444	2.3
	27,682	Balearic Islands	416	2.2
	33,223	Basque Country	788	4.2
	20,892	Canary Islands	838	4.4
	23,757	Cantabria	234	1.2
	24,031	Castile and León	1277	6.7
	20,363	Castilla- La Mancha	824	4.3
	30,426	Catalonia	3036	16.0
	35,041	Community of Madrid	2783	14.7
	18,769	Extremadura	467	2.5
	23,183	Galicia	1083	5.7
	27,225	La Rioja	136	.7
	21,269	Murcia	552	2.9
	31,389	Navarre	214	1.1
	22,426	Valencian Community	1996	10.5

[a]Based on 20 variables, using Multiple Correspondences Analysis (MCA), households were factored into one of four groups as follows:

<u>Low Grade</u>
Head of family:
Owners of agriculture, livestock and fisheries
Employees in agriculture, livestock and fisheries
No studies
Other: students, retired, etc.
Equipment:
No internet
No computer
No mobile phone
No car
No microwave
No vacuum cleaner
No dishwasher
No stereo
1 TV

<u>Medium-Low Grade</u>
Head of family:
Lower levels of employment
Primary school
Equipment:
No dishwasher
1 Bathroom
1 car
1 TV set
No pay TV
No electric toothbrush
No housekeeper
No second residence

Medium-Medium Grade

Head of family:

Mid management
Office employees
Industry and commerce employees
Secondary school

Equipment:

Robotic Vacuum cleaner
Dishwasher
Stereo
2+ cars
2+ TV sets
2+ bathrooms
Electric toothbrush

Medium-High and High Grade

Head of family:

High management
Mid management
Self-employed
University

Equipment:

Housekeeper
Secondary residence
3+ TV sets
2+ cars

Proportion of households purchasing zero- and no-alcohol products and odds ratios.

Dependent variables
The dependent variables were dummy coded yes=1 or no=0 for whether or not an individual household had many at least one purchase of:

- Zero-alcohol beer
- Non-zero no-alcohol beer
- Zero-alcohol wine

Independent variables
The independent variables were dummy-coded for each of the socio-demographic characteristics of the households of Supplement Table 1.

The SPSS syntax was:

```
GENLIN dependent variable (REFERENCE=FIRST) BY community age grade (ORDER=ASCENDING)
  /MODEL community age grade INTERCEPT=YES
 DISTRIBUTION=BINOMIAL LINK=LOGIT
  /CRITERIA METHOD=FISHER(1) SCALE=1 COVB=MODEL MAXITERATIONS=100 MAXSTEPHALVING=5
    PCONVERGE=1E-006(ABSOLUTE) SINGULAR=1E-012 ANALYSISTYPE=3(WALD) CILEVEL=95
CITYPE=WALD
    LIKELIHOOD=FULL
  /EMMEANS TABLES=community SCALE=ORIGINAL
  /EMMEANS TABLES=age SCALE=ORIGINAL
  /EMMEANS TABLES=grade SCALE=ORIGINAL
  /MISSING CLASSMISSING=EXCLUDE
  /PRINT CPS DESCRIPTIVES MODELINFO FIT SUMMARY SOLUTION (EXPONENTIATED).
```

Time Series Analyses

Hypothesis: Increases in purchases of zero-alcohol beers and wines and no-alcohol beers over time are associated with decreased purchases of grams of alcohol.

Sample: 18,954 Spanish households.

The dependent variable is:
1. Grams of all purchased alcohol, per adult per household per day of purchase (primary outcome).

The independent variables are (depending on the model):
- Model 1: Volume of purchases (ml) of zero-alcohol beer; volume of purchases (ml) of no-alcohol beer; volume of purchases (ml) of zero-alcohol wine; ABV of purchases of all other beer; and, ABV of purchases of all other wine;
- Model 2: Same variables for Model 1 plus: volume of purchases (ml) of all other beer; volume of purchases (ml) of all other wine;

Distribution of primary outcome
The primary outcome, grams of all purchased alcohol, per adult per household per day of purchase across all study days was normally distributed, Supplement Figure S1.

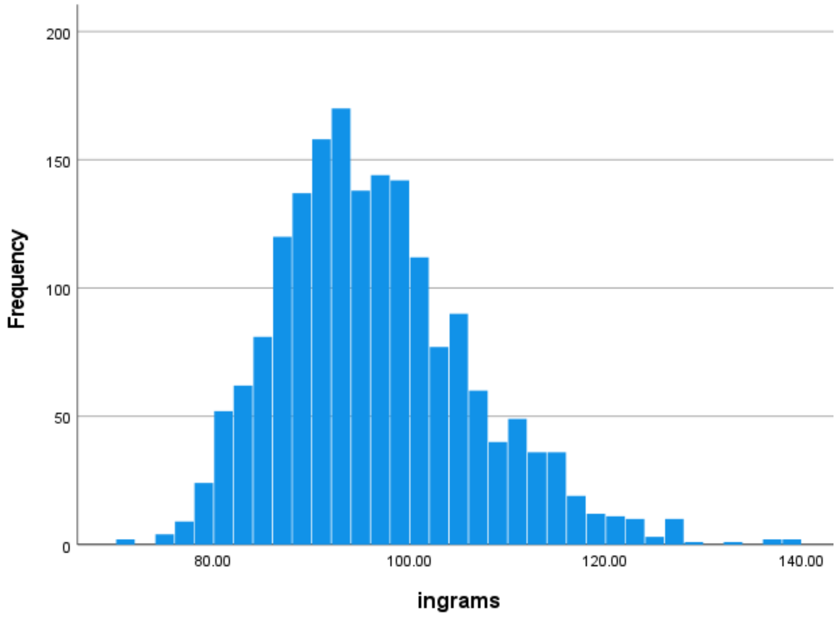

Supplement Figure S1 Frequency distribution of grams of all purchased alcohol per adult per household per day of purchase across all study days (gm).

Seasonal adjustment
To remove annual seasonal variation, we adjusted the data by year using the Census Method I, ratio-to-moving-average method (Makridakis S, Wheelwright SC, McGee VE. *Forecasting: methods and applications*. New York: John Wiley and Sons, 1983; McLaughlin RL. *Forecasting techniques for decision making*. Rockville, MD: Control Data Management Institute, 1984).

Time series
We used a time series modeler function to estimate best fitting non-seasonal and seasonal ARIMA models that: a) specify degrees of differencing and/or a square root or natural log transformation to

ensure a stationary series; and, b) specify autoregressive and moving average orders. This eliminated the need to identify an appropriate ARIMA model through trial and error (ftp://public.dhe.ibm.com/software/analytics/spss/documentation/statistics/26.0/en/client/Manuals/IBM_SPSS_Forecasting.pdf.), with the ARIMA terms (0,1,7) (0,0,0) and residual ACF and PACF plots in Supplementary Figure S2.

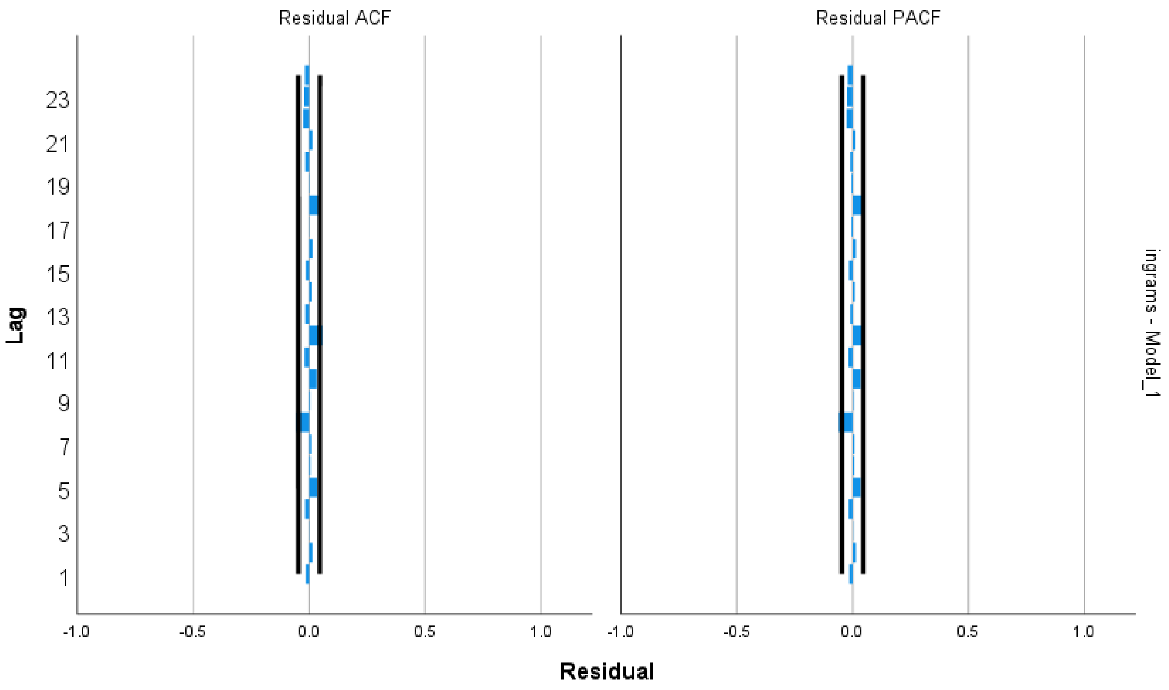

Supplement Figure S2. Residual ACF and PACF of time series for grams of all purchased alcohol per adult per household per day of purchase.

The SPSS Syntax for Model 1 was:

```
TSMODEL
  /MODELSUMMARY  PRINT=[MODELFIT]
  /MODELSTATISTICS  DISPLAY=YES MODELFIT=[ SRSQUARE]
  /MODELDETAILS  PRINT=[ PARAMETERS]  PLOT=[ RESIDACF RESIDPACF]
  /SERIESPLOT OBSERVED FIT
  /OUTPUTFILTER DISPLAY=ALLMODELS
  /AUXILIARY  CILEVEL=95 MAXACFLAGS=24
  /MISSING USERMISSING=EXCLUDE
  /MODEL DEPENDENT=grams INDEPENDENT=zerobml nobml zerowml bbv bwv
    PREFIX='Model'
  /EXPERTMODELER TYPE=[ARIMA EXSMOOTH] TRYSEASONAL=YES
  /AUTOOUTLIER  DETECT=OFF.
```

Where:

- Grams is the number of grams purchased per adult per household per purchase day averaged across all households for each study day

- Zerobml is the volume of purchases (ml) of zero-alcohol beer (ABV=0.0%) per adult per household per purchase day averaged across all households for each study day
- Nobml is the volume of purchases (ml) of non-zero no-alcohol beer (ABV>0.0% and ≤1.0%) per adult per household per purchase day averaged across all households for each study day
- Zerowml is the volume of purchases (ml) of zero-alcohol wine (ABV=0.0%) per adult per household per purchase day averaged across all households for each study day
- BBV is the ABV of purchases of all other beer with an ABV>1.0% per purchase across all averaged across all purchases for each study day
- WBV is the ABV of purchases of all other wine with an ABV>0.0% per purchase across all averaged across all purchases for each study day.

Calculation of price per gram per ABV

For beer with an ABV>3.5% (the cut-off of 3.5% was used to avoid spurious higher costs per gram of alcohol within lower-strength beers), we calculated the cost (€cents) per gram for each purchase of beer, and the cost (€cents) per gram per ABV for each purchase of beer. As these costs were on different scales and, since we were going to compare them, we converted the costs to standardized values, using the SPSS command,

```
DESCRIPTIVES VARIABLES=costpergram costpergramaperABV
  /SAVE
  /STATISTICS=MEAN STDDEV MIN MAX.
```

We plotted the standardized values of the costs against the standardized value of ABV. We used the Curve Estimation procedure of SPSS to determine the best fitting curve of the data (e.g., cubic, quadratic etc), taking that curve with the highest R^2, which was cubic, with the ANOVA coefficients giving us the following equations:

```
Zcost per gram=-0.121+(0.096*zabv)+(0.156*zabv*zabv)-(0.070*zabv*zabv*zabv).
execute.

Zcost per gram per ABV=-0.463-(0.328*zabv)+(0.742*zabv*zabv)-(0.288*zabv*zabv*zabv).
execute.
```

The example SPSS syntax was:

```
CURVEFIT
  /VARIABLES= Zcost per gram WITH zbbv
  /CONSTANT
  /MODEL=CUBIC
  /PRINT ANOVA
  /PLOT FIT
  /SAVE=PRED .
```

Supplement Figure S3, Supplement Page 8 below, plots the predicted standardized values of prices (€cent/g and €cent/g/ABV) from the regression equations against the standardized values of ABV, for beers with an ABV >3.5%. For ABV, the standardized value of 0 is an actual ABV of 7.0 (i.e., a high strength for a beer, with 94.6% of all beer purchases having an ABV ≤7.0%). Prices, expressed as €cent/g demonstrated little variation with ABV, and did not increase with increasing ABV (regression coefficient, price with ABV =0.009, 95%CI=-0.020 to 0.039), whereas prices, adjusted for ABV, decreased with increasing ABV (regression coefficient, price with ABV =-0.634, 95%CI=-0.778 to -0.490). Were prices per gram of alcohol to have increased with increasing ABV, the plot of price expressed as €cent/g/ABV should have remained stable with increasing ABV; thus, the price per gram of alcohol did not increase with increasing strength of ABV.

Supplement Figure S4, Supplement Page 9 below, plots the predicted standardized values of purchases of grams of alcohol by standardized prices (€cent/g and €cent/g/ABV) for beers with an ABV >3.5%. Up to the standardized value of price of 0.0, standardized values of purchased grams of alcohol dropped more steeply for prices expressed as €cent/g/ABV than prices expressed as €cent/g; the coefficient for the interaction term, grams*type of price (€cent/g/ABV or €cent/g) with the standardized value of price was 0.868 (95%CI=0.757 to 0.980) for the drop in purchases with increasing prices, €cent/g/ABV compared to €cent/g. In other words, for every standard deviation increase in

price, purchases decreased by more than 0.87 standard deviations when the price was expressed as €cent/g/ABV than when it was expressed as €cent/g.

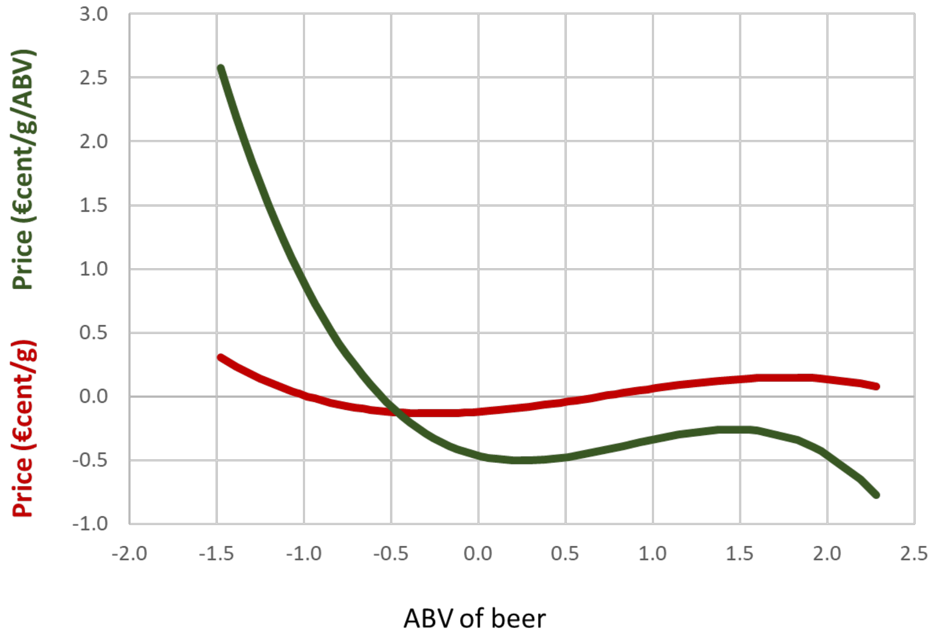

Supplement Figure S3 For purchases of beer with an ABV>3.5%, plots of predicted standardized values of prices (€cent/gram and €cent/gram/ABV) with standardized values of ABV. Data derived from cubic model.

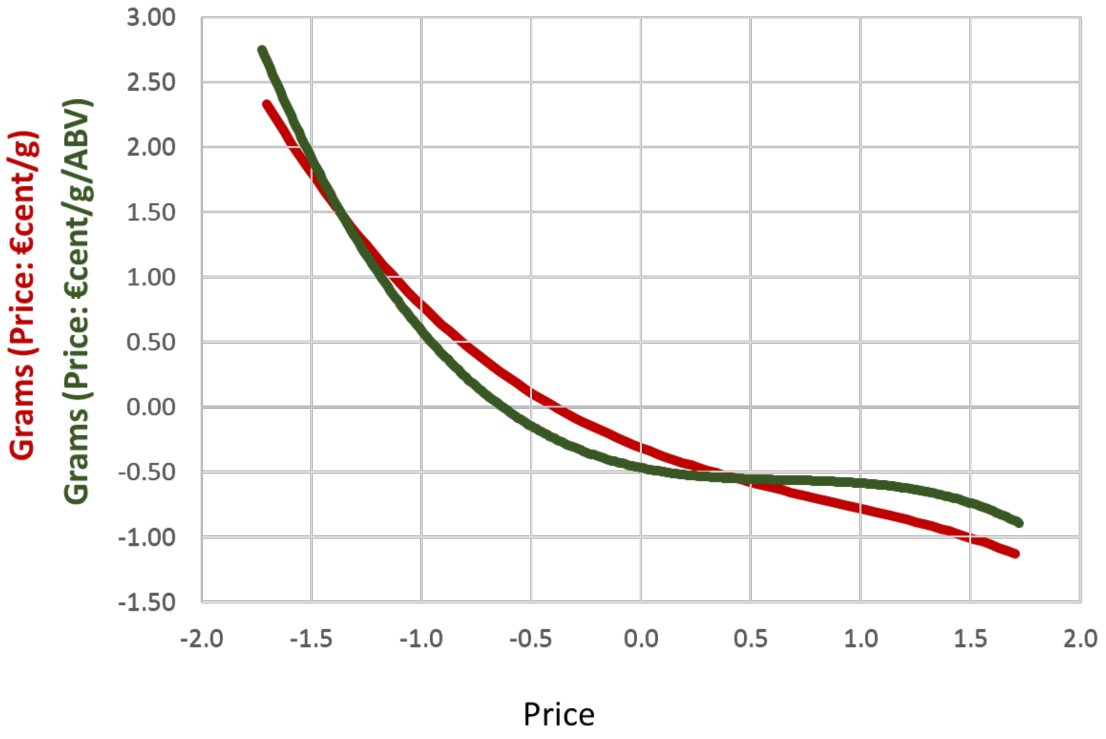

Supplement Figure S4. Plots of predicted standardized purchases of grams of alcohol within beer for beer with an ABV>3.5% by standardized price of beer, for when price calculated as €cent per gram of alcohol with beer purchased and as €cent per gram of alcohol within beer purchased per ABV of beer. Data derived from cubic model.

Time Series Analyses for price.

With the same primary outcome (dependent variable) as above, grams of alcohol purchased, the SPSS syntax is:

```
TSMODEL
  /MODELSUMMARY  PRINT=[MODELFIT]
  /MODELSTATISTICS  DISPLAY=YES MODELFIT=[ SRSQUARE]
  /MODELDETAILS  PRINT=[ PARAMETERS]  PLOT=[ RESIDACF RESIDPACF]
  /SERIESPLOT OBSERVED FIT
  /OUTPUTFILTER DISPLAY=ALLMODELS
  /AUXILIARY  CILEVEL=95 MAXACFLAGS=24
  /MISSING USERMISSING=EXCLUDE
  /MODEL DEPENDENT=grams afbeerml bbv independent =afbp beerp beerpabv
    PREFIX='Model'
  /EXPERTMODELER TYPE=[ARIMA EXSMOOTH] TRYSEASONAL=YES
  /AUTOOUTLIER  DETECT=OFF.
```

Where:

- Grams is the number of grams purchased per adult per household per purchase day averaged across all households for each study day
- BBV is the ABV of purchases of all other beer with an ABV>3.5% per purchase across all averaged across all purchases for each study day
- Afbeerml is the volume of purchases (ml) of no-alcohol beer (ABV≤1.0%) per adult per household per purchase day averaged across all households for each study day
- Beerp is the price per gram of alcohol within beer (with an ABV>3.5%) per purchase averaged across all purchases for each study day.
- Beerpabv is the price per gram of alcohol within beer (with an ABV>3.5%) per ABV per purchase averaged across all purchases for each study day.

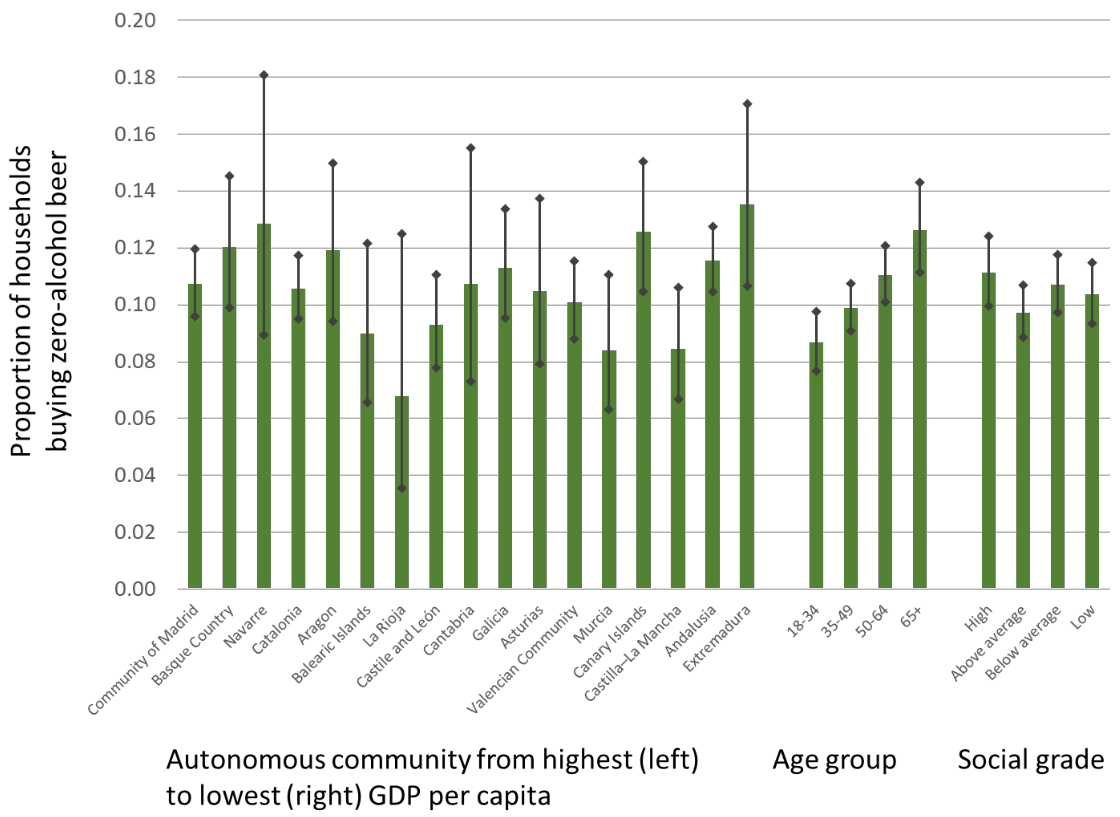

Supplement Figure S5. Proportion of households (whiskers=95% confidence intervals) making at least one purchase of zero-alcohol beer by household attributes.

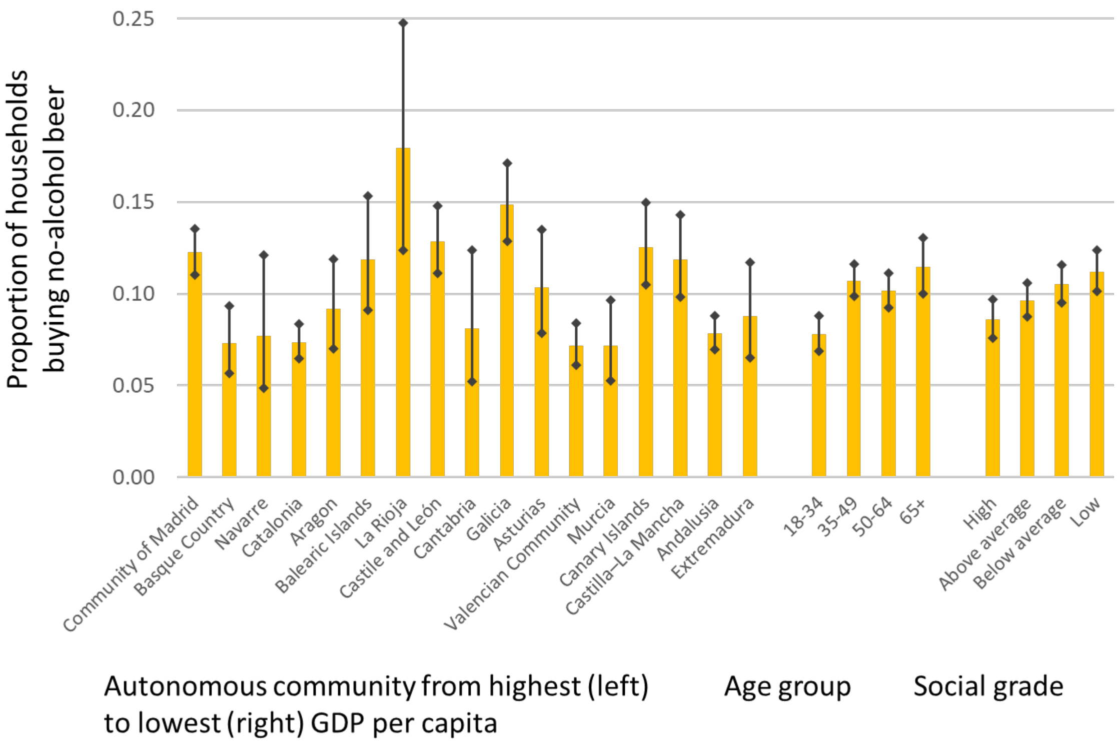

Supplement Figure S6. Proportion of households (whiskers=95% confidence intervals) making at least one purchase of no-alcohol beer by household attributes.

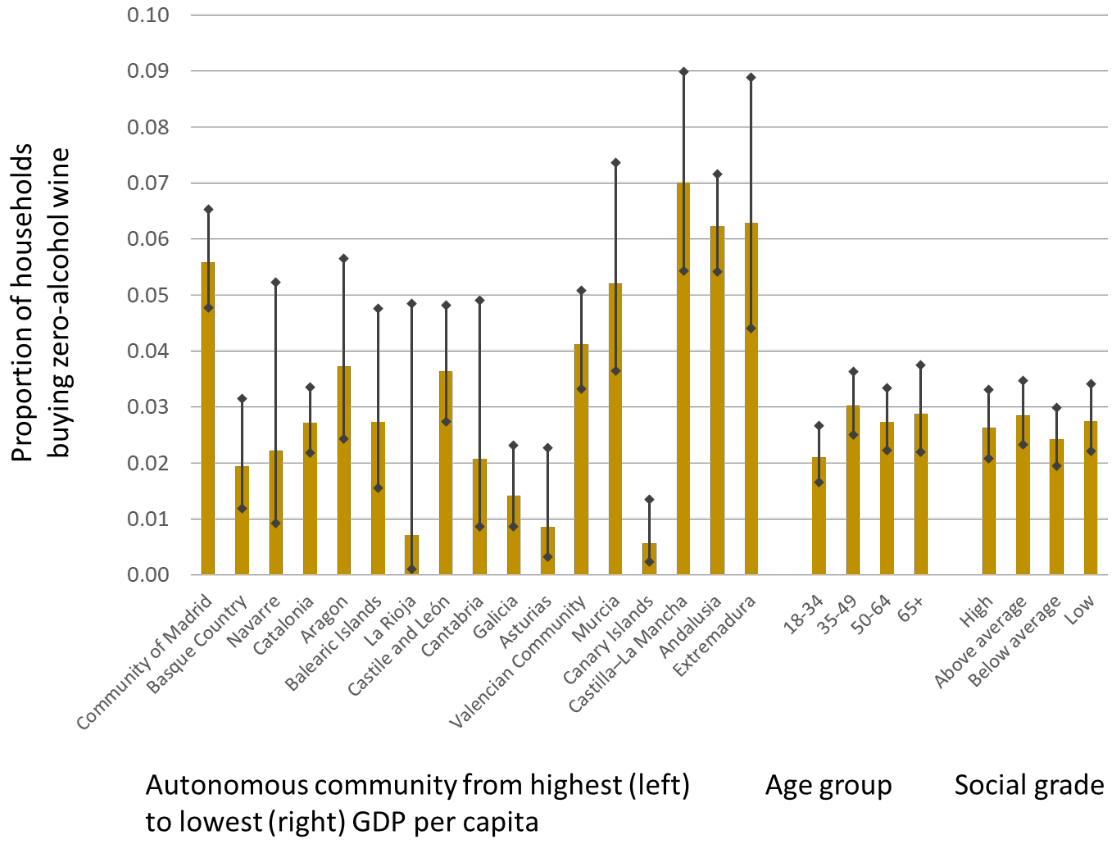

Supplement Figure S7. Proportion of households (whiskers=95% confidence intervals) making at least one purchase of zero-alcohol wine by household attributes.

Supplement Table S2 Proportion of households (and odds ratios) making at least one purchase of zero-alcohol beer, non-zero-no-alcohol beer and zero-alcohol wine by household characteristics.

	**Zero-alcohol beer**		**Non-zero-no-alcohol beer**		**Zero-alcohol wine**	
	Proportion (95% confidence interval)	Odds ratio (95% confidence interval)	Proportion (95% confidence interval)	Odds ratio (95% confidence interval)	Proportion (95% confidence interval)	Odds ratio (95% confidence interval)
**Autonomous community (listed from highest to lowest GDP per capita)**						
Community of Madrid	.107 (.096 to .120)	.768 (.570 to 1.034)	.122 (.110 to .135)	1.454 (1.035 to 2.045)	.056 (.048 to .065)	.882 (.589 to 1.321)
Basque Country	.120 (.099 to .145)	.873 (.617 to 1.236)	.073 (.057 to .093)	.820 (.541 to 1.244)	.019 (.012 to .031)	.294 (.158 to .546)
Navarre	.128 (.089 to .181)	.941 (.578 to 1.531)	.077 (.048 to .121)	.872 (.483 to 1.575)	.022 (.009 to .052)	.338 (.129 to .885)
Catalonia	.106 (.095 to .117)	.755 (.562 to 1.015)	.074 (.065 to .083)	.827 (.584 to 1.172)	.027 (.022 to .034)	.415 (.271 to .638)
Aragon	.119 (.094 to .150)	.865 (.593 to 1.262)	.092 (.070 to .119)	1.050 (.682 to 1.617)	.037 (.024 to .057)	.576 (.325 to 1.021)
Balearic Islands	.090 (.066 to .121)	.629 (.408 to .971)	.119 (.091 to .153)	1.403 (.908 to 2.168)	.027 (.016 to .048)	.419 (.211 to .830)
La Rioja	.068 (.036 to .125)	.464 (.224 to .961)	.179 (.124 to .252)	2.279 (1.327 to 3.912)	.007 (.001 to .048)	.106 (.014 to .785)
Castile and León	.093 (.078 to .111)	.655 (.470 to .913)	.128 (.111 to .148)	1.536 (1.071 to 2.202)	.036 (.027 to .048)	.563 (.352 to .901)
Cantabria	.107 (.073 to .155)	.769 (.465 to 1.270)	.081 (.052 to .124)	.920 (.521 to 1.625)	.021 (.009 to .049)	.316 (.121 to .826)
Galicia	.113 (.095 to .134)	.815 (.585 to 1.135)	.149 (.129 to .171)	1.822 (1.269 to 2.615)	.014 (.009 to .023)	.215 (.116 to .398)
Asturias	.105 (.079 to .137)	.748 (.497 to 1.126)	.103 (.078 to .135)	1.202 (.773 to 1.868)	.009 (.003 to .023)	.129 (.045 to .371)
Valencian Community	.101 (.088 to .115)	.717 (.526 to .976)	.072 (.061 to .084)	.805 (.560 to 1.156)	.041 (.033 to .051)	.640 (.416 to .983)
Murcia	.084 (.063 to .110)	.585 (.389 to .880)	.072 (.053 to .096)	.804 (.510 to 1.266)	.052 (.036 to .074)	.818 (.485 to 1.379)
Canary Islands	.126 (.105 to .150)	.919 (.654 to 1.292)	.125 (.105 to .150)	1.496 (1.024 to 2.184)	.006 (.002 to .014)	.085 (.033 to .220)
Castilla–La Mancha	.084 (.067 to .106)	.589 (.407 to .853)	.119 (.098 to .143)	1.406 (.957 to 2.064)	.070 (.054 to .090)	1.123 (.712 to 1.770)
Andalusia	.116 (.105 to .127)	.835 (.623 to 1.118)	.078 (.069 to .088)	.885 (.627 to 1.250)	.062 (.054 to .072)	.990 (.667 to 1.470)
Extremadura (reference category)	.135 (.106 to .170)	1.000 (. to .)	.087 (.065 to .117)	1.000 (. to .)	.063 (.044 to .089)	1.000 (. to .)
**Age group of main household shopper**						
18-34	.087 (.077 to .097)	.655 (.549 to .782)	.078 (.069 to .088)	.653 (.543 to .787)	.021 (.017 to .027)	.726 (.543 to .969)
35-49	.099 (.091 to .107)	.759 (.651 to .885)	.107 (.099 to .116)	.928 (.791 to 1.090)	.030 (.025 to .036)	1.053 (.819 to 1.354)
50-64	.110 (.101 to .121)	.859 (.734 to 1.005)	.101 (.093 to .111)	.874 (.740 to 1.033)	.027 (.022 to .033)	.950 (.730 to 1.236)
65+ (reference category)	.126 (.111 to .143)	1.000 (. to .)	.114 (.100 to .130)	1.000 (. to .)	.029 (.022 to .037)	1.000 (. to .)
**Social grade of household**						
Medium-High and High	.111 (.099 to .124)	1.084 (.933 to 1.258)	.086 (.076 to .097)	.743 (.634 to .871)	.026 (.021 to .033)	.958 (.759 to 1.209)
Medium-Medium	.097 (.088 to .107)	.932 (.816 to 1.065)	.096 (.088 to .106)	.844 (.739 to .964)	.028 (.023 to .035)	1.038 (.852 to 1.266)
Medium-Low	.107 (.097 to .118)	1.037 (.906 to 1.187)	.105 (.095 to .115)	.929 (.811 to 1.065)	.024 (.020 to .030)	.878 (.710 to 1.087)
Low (reference category)	.104 (.093 to .115)	1.000 (. to .)	.112 (.101 to .124)	1.000 (. to .)	.027 (.022 to .034)	1.000 (. to .)

Supplement Table S3 Regression coefficients for changes over time for stated dependent variables; independent variable time, study day from day 114 to day 1933.

	Zero-beer	Non-zero no-alcohol beer	Zero-wine	Beer ABV	Wine ABV	Grams all alcohol
Intercept	92.8038 (91.8729 to 93.7346)	97.6628 (96.5873 to 98.7383)	32.7451 (32.2695 to 33.2208)	5.0144 (5.0114 to 5.0173)	12.2079 (12.2027 to 12.2130)	92.5802 (92.1290 to 93.0314)
Time (unit=100 days)	1.1758 (1.0952 to 1.2565)	-1.0459 (-1.1391 to -.9527)	-.3796 (-.4209 to -.3384)	.0036 (.0033 to .0038)	.0073 (.0068 to .0077)	.4010 (.3618 to .4401)

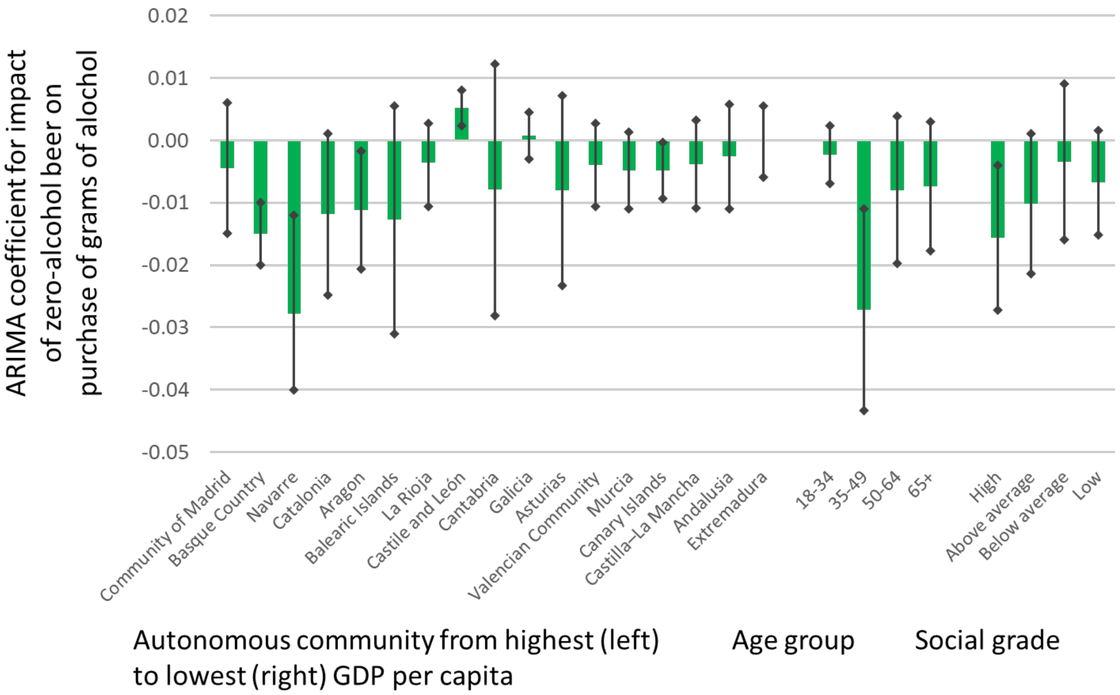

Supplement Figure S8. ARIMA coefficients for the associations between changes in purchase of zero-alcohol beer and changes in purchase of all grams of alcohol by each of the socio-demographic attributes of the household.

Supplement Table S4 Regression coefficients for changes over time for stated variables; independent variable time, study day from day 114 to day 1933.

	**Price no-alcohol beer (ABV≤1.0%) (€/L)**	**Price all other beer (ABV>3.5%) (€cent/g)**	**Price all other beer (ABV>3.5%) (€cent/g/ABV)**	**Volume no-alcohol beer (ABV≤1.0%) (dl)**	**ABV other beer (for beers with ABV>3.5%)**	**Grams all alcohol (hectograms)**
Intercept	1.269 (1.267 to 1.270)	3.377 (3.370 to 3.384)	0.659 (0.658 to 0.660)	1.895 (1.894 to 1.896)	5.01 (5.01 to 5.02)	0.924 (0.921 to 0.928)
Time (unit=100 days)	12.48^{-3} (12.32^{-3} to 12.64^{-3})	3.09^{-4} (3.03^{-4} to 3.15^{-4})	5.36^{-5} (5.27^{-5} to 5.45^{-5})	2.11^{-3} (2.01^{-3} to 2.20^{-3})	3.42^{-3} (3.29^{-3} to 3.56^{-3})	4.16^{-3} (3.83^{-3} to 4.84^{-3})
